# Myeloid Heme Oxygenase-1 Regulates the Acute Inflammatory Response to Zymosan in the Mouse Air Pouch

**DOI:** 10.1155/2018/5053091

**Published:** 2018-02-11

**Authors:** Rita Brines, Laura Catalán, Maria José Alcaraz, Maria Luisa Ferrándiz

**Affiliations:** Instituto Interuniversitario de Investigación de Reconocimiento Molecular y Desarrollo Tecnológico (IDM), Universitat Politècnica de València, Universitat de València, Av. Vicent A. Estellés, s/n, Burjassot, 46100 Valencia, Spain

## Abstract

Heme oxygenase-1 (HO-1) is induced by many stimuli to modulate the activation and function of different cell types during innate immune responses. Although HO-1 has shown anti-inflammatory effects in different systems, there are few data on the contribution of myeloid HO-1 and its role in inflammatory processes is not well understood. To address this point, we have used HO-1^M-KO^ mice with myeloid-restricted deletion of HO-1 to specifically investigate its influence on the acute inflammatory response to zymosan *in vivo*. In the mouse air pouch model, we have shown an exacerbated inflammation in HO-1^M-KO^ mice with increased neutrophil infiltration accompanied by high levels of inflammatory mediators such as interleukin-1*β*, tumor necrosis factor-*α*, and prostaglandin E_2_. The expression of the degradative enzyme matrix metalloproteinase-3 (MMP-3) was also enhanced. In addition, we observed higher levels of serum MMP-3 in HO-1^M-KO^ mice compared with control mice, suggesting the presence of systemic inflammation. Altogether, these findings demonstrate that myeloid HO-1 plays an anti-inflammatory role in the acute response to zymosan *in vivo* and suggest the interest of this target to regulate inflammatory processes.

## 1. Introduction

Heme oxygenase (HO) catalyzes the oxidative degradation of heme to carbon monoxide (CO), iron, and biliverdin which is converted to bilirubin by biliverdin reductase [1]. HO-2 is constitutively expressed in the testes, brain, and endothelium and would regulate normal physiological functions while HO-1 is highly inducible by a wide range of stimuli. HO-1 plays an important role in the antioxidant defence system and iron homeostasis. In addition, HO-1 is involved in the regulation of different cell functions such as proliferation, differentiation, and apoptosis (reviewed in [2]). A wide range of evidence indicate that HO-1 regulates the activation and function of different cell types driving the inflammatory process and innate and adaptive immune responses [3–8]. Therefore, HO-1 upregulation or administration of its metabolites results in anti-inflammatory and antioxidant effects in many disease models such as atherosclerosis [9], cardiac ischemia/reperfusion injury [10], diabetes [11], inflammatory bowel disease [12], or rheumatoid arthritis [4]. Conversely, the deletion of *Hmox1* in mice increases the severity of many experimental diseases, and the incidence or severity of several human diseases are associated with polymorphisms in the *Hmox1* promoter that regulates HO-1 expression [7, 13].

HO-1 knockout (*Hmox1*^−/−^) mice exhibit growth retardation, anemia, proteinuria, hepatic and renal iron accumulation, chronic inflammation, and reduced life span [14], which are similar to the important alterations observed in human HO-1 deficiency [15, 16]. Conditional deletion of HO-1 could allow a more specific approach to study the role of HO-1 in physiological and pathological processes. Previous works have shown that mice with selective deletion of myeloid HO-1 (HO-1^M-KO^) do not exhibit any relevant physiologic changes compared with wild-type animals, and unlike HO-1^−/−^ mice, they do not have enlarged spleens or pathological abnormalities [17].

A great number of studies have been devoted to assess HO-1 biological effects, in contrast with the relatively few investigations using targeted approaches to specifically focus on myeloid HO-1. Previous reports have indicated that myeloid HO-1 may be a protective pathway against renal ischemia/reperfusion injury [18] or autoimmune central nervous system diseases. Therefore, genetic ablation of myeloid HO-1 enhances the infiltration of macrophages and Th17 cells into the central nervous system resulting in aggravation of experimental autoimmune encephalomyelitis [17]. In contrast, other works have shown that macrophage conditional HO-1 deletion evokes resistance to inflammation and metabolic disease [19], and myeloid HO-1 would not play a main role in lung inflammation induced by lipopolysaccharide [20]. It is therefore apparent that more studies are necessary to understand the role of myeloid HO-1 in inflammatory conditions.

Zymosan particles are recognized by the innate immune system and induce inflammatory signals through Toll-like receptors TLR2 and TLR6 leading to the production of a variety of inflammatory mediators [21]. The zymosan-induced mouse air pouch (MAP) is a widely used experimental model useful to dissect the inflammatory response. Besides, myeloid cells play an important role in zymosan-induced inflammation which makes this stimulus appropriate to characterize the contribution of myeloid HO-1. The aim of this work was to investigate *in vivo* whether myeloid HO-1 deletion could affect key steps of the early inflammatory response in the zymosan-induced MAP.

## 2. Materials and Methods

### 2.1. Animals

All studies were performed in accordance with European Union regulations for the handling and use of laboratory animals. All the animal experiments were approved by the Institutional Animal Care and Use Committee of the University of Valencia, Spain. Mice were housed and cared for by the veterinary staff in accredited facilities and were routinely screened for health status. *Hmox1*^FL/FL^ (129.B6-Hmox1tm1.1Gkl/FlmgEM: 10935) mice were obtained from Alexander Fleming Biomedical Sciences Research Center, Vari, Greece, and crossed with *LysM*^Cre^ (B6.129P2-Lyz2tm1(cre)Ifo/CgnCnrm EM:01145) mice from EMMA (European Mouse Mutant Archive) Repository, Istituto Biologia Cellulare CNR, Monterotondo Scalo, Italy, and MGC Stiftung (Mouse Genetics Cologne (MGC) Foundation, Munich, Germany). Genotyping of mice was performed using the KAPA Mouse Genotyping Kit (KAPA Biosystems, Boston, MA, USA). Primers used for real-time PCR were as follows: *Hmox1* (F 5′-AGGGGAAGAAGACTCATCGA-3′, R 5′-AGAAGGCTCGGAGGTTAAAT-3′), *LysM*^Cre^ (F 5′-CGAGTGATGAGGTTCGCAAG-3′, R 5′-TGAGTGAACGAACCTGGTCG-3′), and *LysM*^WT^ (F 5′-GCATTGCAGACTAGCTAAAGGCAG-3′, R 5′-GTCGGCCAGGCTGACTCCATAG-3′). For *Hmox1*, 1 cycle was for 4 min in 94°C; 1 cycle for 45 s*g* in 52°C; 30 cycles for 45 s*g* in 93°C, 45 s*g* in 52°C, and 1 min in 72°C; and 1 cycle for 10 min in 72°C. For *Cre*, 1 cycle was for 2 min in 95°C; 45 cycles for 30 s*g* in 95°C, 30 s*g* in 58°C, 90 s*g* in 72°C; and 1 cycle for 10 min in 72°C. The products, 545 bp (*Hmox1*^FL^), 387 bp (*Hmox1*^WT^), 390 bp (*LysM*^Cre^), and 428 bp (*LysM*^WT^) were analyzed with the Agilent Bioanalyzer 2100 (Agilent Technologies Spain, Madrid). To confirm the suppression of HO-1, we isolated peritoneal macrophages from *LysM*^Cre/+^*Hmox1*^FL/FL^ (HO-1^M-KO^) and *Hmox1*^FL/FL^ (control) mice and analyzed HO-1 expression by immunocytochemistry and flow cytometry. Macrophages were obtained by peritoneal lavage with saline. HO-1 expression was assessed in adherent cells stimulated with 0.1 *μ*g/ml phorbol 12-myristate 13-acetate (Sigma-Aldrich, St. Louis, MO, USA) for 18 h at 37°C. Cells were fixed with 2% paraformaldehyde (5 min, 4°C) and permeabilized with 0.1% Triton X-100 for 10 min. After blocking (10% bovine serum albumin), anti-HO-1 polyclonal antibody (Enzo Life Sciences Inc.) was incubated overnight at 4°C. Unbound antibody was removed with phosphate-buffered saline, and the secondary fluorescent antibody (anti-rabbit IgG, R&D Systems, Minneapolis, MN, USA) was incubated for 30 min. Macrophages were observed with a fluorescence microscope (Leica DM IL LED, Solms, Germany). Cells were also analyzed by flow cytometry with the anti-mouse CD16/CD32 antibody for blockade (eBioscience, Thermo Fisher Scientific, Waltham, MA, USA) followed by anti-F4/80-APC, anti-Ly-6G (Gr-1)-PE (eBioscience, Thermo Fisher Scientific), and HO-1-FITC mAb (Enzo Life Sciences Inc.) using FACScan and Cell Quest Pro (Becton Dickinson, Madrid, Spain).

### 2.2. Mouse Air Pouch (MAP)

Mice were maintained at 21°C ± 2°C on a 12 h light/dark cycle with feed and water ad libitum. Male HO-1^M-KO^ and control mice between 10 and 12 weeks were used in these experiments. To create the MAP, 10 ml of sterile air was injected subcutaneously into the dorsal area of the mouse (day 0). Three days later, 5 ml of sterile air was injected (day 3), and six days after the initial injection of sterile air (day 6), 1 ml of saline buffer or 1 ml of 1% *w*/*v* zymosan (Sigma-Aldrich) in saline buffer was injected into the air pouch [22]. At 18 h after zymosan stimulation, blood was collected from the retroorbital venous plexus, mice were sacrificed by cervical dislocation, and the exudate of the air pouch was collected. Cells present in exudates were measured with a Coulter counter and differential counting was also performed. Exudates were centrifuged at 10,000 ×g for 5 min at 4°C. Supernatants were then collected and frozen at −80°C to measure the levels of inflammatory and catabolic mediators. Cell pellets were lysed and used for HO-1 determination by western blotting.

### 2.3. Determination of Inflammatory Mediators

Interleukin-1*β* (IL-1*β*) and tumor necrosis factor-*α* (TNF*α*) were measured by enzyme-linked immunosorbent assay (ELISA) (R&D Systems, Minneapolis, MN, USA) with a range detection of 15.6–1000 pg/ml and 31.2–2000 pg/ml, respectively. C-X-C motif chemokine ligand 1 (CXCL-1) ELISA was from PromoKine (Heidelberg, Germany) with a sensitivity of 8.0 pg/ml. MMP-3 levels were measured by ELISA (R&D Systems Europe Ltd., Abingdon, UK), with sensitivity of 19.0 pg/ml. The eicosanoid prostaglandin E_2_ (PGE_2_) was determined by radioimmunoassay [23].

### 2.4. Western Blotting

Cell pellets from the air pouch exudates were resuspended in a buffer pH 7.46 containing 10 mM HEPES, 1 mM EDTA, 1 mM EGTA, 10 mM KCl, 1 mM dithiothreitol, 5 mM NaF, 1 mM Na_3_VO_4_, 1 *μ*g/ml leupeptin, 0.1 *μ*g/ml aprotinine, and 0.5 mM phenylmethyl sulfonyl fluoride and were sonicated (3 × 10 sec) and centrifuged at 10,000 ×g for 10 min at 4°C. Supernatants were collected and protein concentration was evaluated by the DC protein reagent (Bio-Rad, Hercules, CA, USA). Proteins (30 *μ*g) were separated by SDS/PAGE (12.5%) and transferred to PVDF membranes. Membranes were blocked with 5% nonfat dry milk and incubated with specific polyclonal antibody against HO-1 (1/1000) (Enzo Life Sciences Inc., Farmingdale, NY, USA) or *β*-actin (1/5000, Sigma-Aldrich) for 2 h at room temperature. Finally, membranes were incubated with peroxidase-conjugated polyclonal goat anti-rabbit IgG (1/5000, Dako, Glostrup, Denmark) and the immunoreactive bands were visualized by enhanced chemiluminescence (ECL®, GE Healthcare, Buckinghamshire, UK) using an AutoChemi image analyser (UVP Inc., Upland, CA, USA). Band intensity was analysed by optical densitometry using ImageJ analysis software (NIH, USA). Band densities were corrected for background, and protein levels were normalized against *β*-actin.

### 2.5. Statistical Analysis

The data were presented as mean ± SD. Normal distribution of data was assessed by the Shapiro-Wilk normality test. Two-way analysis of variance (ANOVA) followed by Sidak's multiple comparisons test, or unpaired *t*-test was performed. All statistical analyses were performed using GraphPad Prism version 7.00 for Windows (GraphPad Software, San Diego, CA, USA). A *P* value lower than 0.05 was considered to indicate statistical significance.

## 3. Results

### 3.1. Confirmation of HO-1 Deletion in Mouse Peritoneal Macrophages

**Figure 1:**
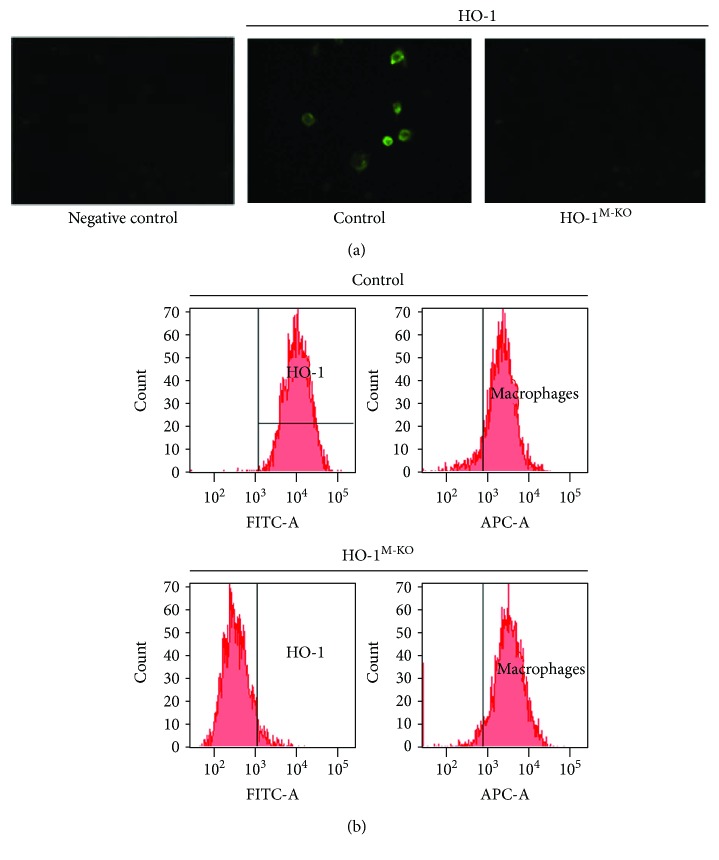
Expression of HO-1 in peritoneal macrophages from HO-1^M-KO^ and control mice. (a) Immunocytochemistry for HO-1 expression. Representative images. (b) Flow cytometry. F4/80-positive cells expressed HO-1 in control mice but not in HO-1^M-KO^ mice.

*Hmox1* ^FL/FL^ mice were crossed with *LysM*^Cre^ knockin mice for deletion of HO-1 in myeloid cells. The *LysM*^Cre^ transgene mediates the excision of loxP-flanked sequences in these cells [24]. We assessed HO-1 protein expression in mouse peritoneal macrophages by immunocytochemistry (Figure 1(a)) and flow cytometry (Figure 1(b)). HO-1 expression was observed in macrophages from *Hmox1*^FL/FL^ (control) but not in *LysM*^Cre/+^*Hmox1*^FL/FL^ (HO-1^M-KO^) mice, thus confirming the efficient deletion of myeloid HO-1.

### 3.2. Cell Migration into the MAP

**Figure 2:**
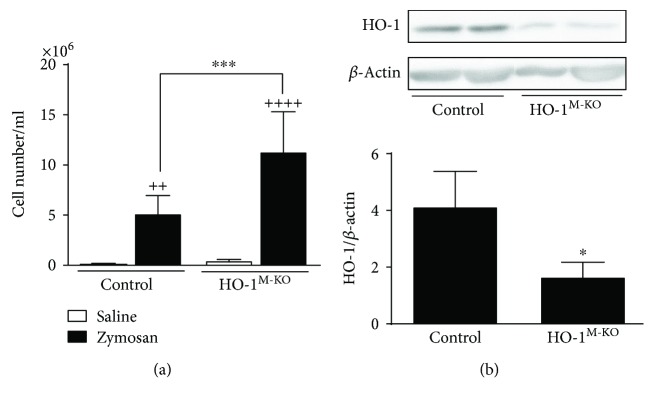
Cell migration into the MAP. (a) Cell numbers in the exudate, mean ± SD (*n* = 6). (b) Immunoblot for HO-1 expression in cells of the exudate. Representative images and rate of HO-1/*β*-actin; ^++^*P* < 0.01 and ^++++^*P* < 0.0001 with respect to saline-injected group; ^∗^*P* < 0.05 and ^∗∗∗^*P* < 0.001, compared to control mice (a: two-way ANOVA followed by Sidak's test; b: unpaired *t*-test).

Zymosan induces cell activation and production of a wide range of proinflammatory mediators that are quickly released into the inflammatory milieu. As a consequence, there is an important leukocyte influx into the air pouch. We have previously reported that migrating cells are predominantly neutrophils. They represent about 88-89% of cells present in air pouch exudates [25]. The interaction between treatment (saline or zymosan) and genotype (control or HO-1^M-KO^) was significant for cell migration (*F* = 10.67, *P* = 0.0039).  Figure 2(a) shows that zymosan induced a significant cell migration into the air pouch compared with saline-injected air pouches in both control and HO-1^M-KO^ mice. It is interesting to note that inflammatory cell accumulation in air pouch exudate induced by zymosan was significantly higher in HO-1^M-KO^ mice compared with control mice (11.3 ± 3.9 versus 5.1 ± 1.7 × 10^6^ cells/ml). In this experimental model, there is a time-dependent increase in HO-1 expression in the cells migrating to the air pouch exudate [26]. We assessed HO-1 protein expression in these migrating cells and observed a high level in cells from control animals whereas a very reduced expression was present in cells from HO-1^M-KO^ mice (Figure 2(b)). HO-1 protein was not completely suppressed in this last group as some nonmyeloid cells are also present in exudates [25].

### 3.3. Proinflammatory Mediators in Air Pouch Exudate

**Figure 3:**
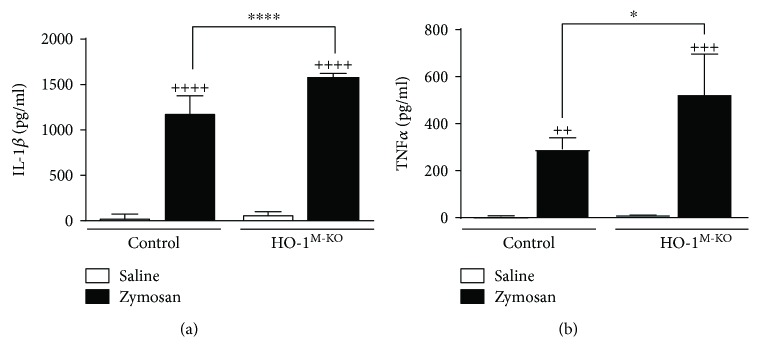
Levels of proinflammatory cytokines in MAP exudate. IL-1*β* (a) and TNF*α* (b) were measured by ELISA. Results are shown as mean ± SD (*n* = 6); ^++^*P* < 0.01, ^+++^*P* < 0.001, and ^++++^*P* < 0.0001 with respect to saline-injected group; ^∗^*P* < 0.05 and ^∗∗∗∗^*P* < 0.0001 compared to control mice (two-way ANOVA followed by Sidak's test).

**Figure 4:**
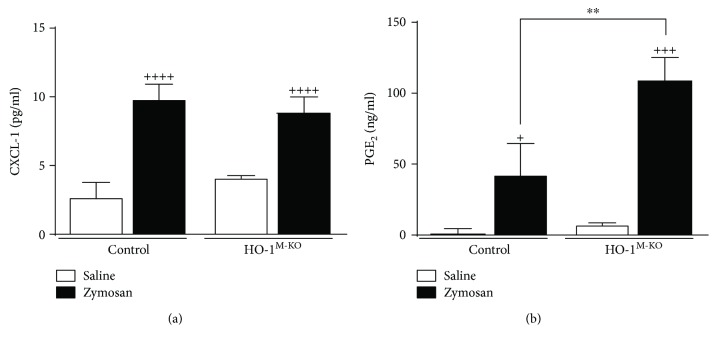
Levels of CXCL-1 and PGE_2_ in the MAP exudate. CXCL-1 (a) was measured by ELISA. PGE_2_ (b) was measured by radioimmunoassay. Results are shown as mean ± SD (*n* = 6); ^+^*P* < 0.05, ^+++^*P* < 0.001, and ^++++^*P* < 0.0001 with respect to saline-injected group; ^∗∗^*P* < 0.01 compared to control mice (two-way ANOVA followed by Sidak's test).

There were no significant differences in levels of proinflammatory mediators between control and HO-1^M-KO^ mice in the absence of zymosan stimulation. The proinflammatory cytokines IL-1*β* and TNF*α* play an important role in the response induced by zymosan (Figures 3(a) and 3(b)). Our results indicate that IL-1*β* and TNF*α* production in response to zymosan depended on genotype (*F* = 19.36, *P* = 0.0003 and *F* = 5.27, *P* = 0.0472, resp.). Zymosan elicited a higher IL-1*β* level in HO-1^M-KO^ mice (1582.0 ± 40.3 pg/ml) compared with control mice (1185.0 ± 191.1 pg/ml). Similarly, TNF*α* levels in the presence of zymosan were enhanced in HO-1^M-KO^ mice (521.1 ± 175.0 pg/ml) with respect to control animals (290.8 ± 49.02 pg/ml). In addition, the interaction between treatment and genotype was significant for the production of the chemokine CXCL-1 (*F* = 8.13, *P* = 0.0102). The levels of CXCL-1 in inflammatory exudates were increased by zymosan administration in both control and HO-1^M-KO^ mice (Figure 4(a)) although there was no significant difference between them (9.83 ± 1.0 and 8.9 ± 1.0 pg/ml, resp.). PGE_2_ is an important proinflammatory mediator in this experimental model. We found that PGE_2_ production induced by zymosan depended on genotype (*F* = 12.11, *P* = 0.0083).  Figure 4(b) shows that zymosan stimulation resulted in high levels of this eicosanoid in the inflammatory exudate in both control and HO-1^M-KO^ mice. The production of PGE_2_ in HO-1^M-KO^ mice (109.0 ± 16.1 ng/ml) was significantly higher than that of control animals (42.7 ± 21.9 ng/ml).

### 3.4. Levels of MMP-3 in Air Pouch Exudate and Serum

**Figure 5:**
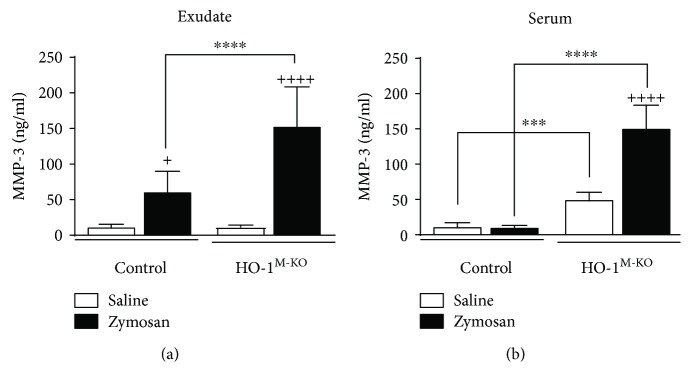
Levels of MMP3 in the MAP exudate (a) and serum (b). MMP-3 was measured by ELISA. Results are shown as mean ± SD (*n* = 6); ^+^*P* < 0.05 and ^++++^*P* < 0.0001 with respect to saline-injected group; ^∗∗∗^*P* < 0.001 and ^∗∗∗∗^*P* < 0.0001 compared to control mice (two-way ANOVA followed by Sidak's test).

There was a significant interaction between treatment and genotype for MMP-3 levels in air pouch exudate and serum (*F* = 16.75, *P* = 0.0006 and *F* = 58.93, *P* < 0.0001, resp.). MMP-3 levels in the air pouch exudate were enhanced by the inflammatory response to zymosan (Figure 5(a)), and we observed a stronger effect in HO-1^M-KO^ mice (153.5 ± 54.8 ng/ml) compared with controls (61.3 ± 28.6 ng/ml). We also assessed MMP-3 levels in serum as a marker of systemic inflammation. As shown in Figure 5(b), in the absence of zymosan stimulation, serum levels of MMP-3 were significantly higher in HO-1^M-KO^ mice (50.1 ± 9.9 versus 11.6 ± 5.4 ng/ml). Zymosan injection did not increase serum MMP-3 in the control group (10.6 ± 2.5 ng/ml) but exerted a dramatic effect in HO-1^M-KO^ mice (151.2 ± 32.5 ng/ml).

## 4. Discussion

Myeloid cells have important roles in both innate and adaptive immune responses [27]. Studies in mice with specific deletion of HO-1 in myeloid cells have revealed the contribution of HO-1 to the maturation and differentiation of monocytes/macrophages. In addition, HO-1 is relevant for cell functions such as phagocytic activity and bacterial clearance. These actions of HO-1 can be reproduced by exogenously applied CO [28, 29] which exerts anti-inflammatory and proresolution effects [30]. We have demonstrated using HO-1^M-KO^ mice that myeloid-restricted deletion of HO-1 potentiates the inflammatory response to zymosan. These results are consistent with our previous data showing that induction of HO-1 exerts anti-inflammatory effects in this experimental model [26]. In the current study, we have shown that HO-1^M-KO^ mice exhibit increased neutrophil infiltrates in response to zymosan administration. This effect on cell migration was higher than that observed in another inflammatory model using HO-1^+/−^ and HO-1^−/−^ mice [13]. Our results thus indicate that myeloid HO-1 plays an important role in the regulation of neutrophil migration and the production of inflammatory and catabolic mediators during the acute response to zymosan *in vivo*. In particular, we observed an increased production of IL-1*β*, TNF*α*, PGE_2_, and MMP-3 in the inflammation focus. This is in contrast to the observed in vitro response of mouse macrophages to lipopolysaccharide, where myeloid HO-1 is not essential for TLR4-induced nuclear factor-*κ*B activation and proinflammatory cytokine production but is required for TLR4/TLR3/interferon regulatory factor 3-induced production of interferon-*β* [17].

Proinflammatory cytokines and elevated levels of PGE_2_ promote the production of MMPs. These enzymes degrade extracellular matrix but they also contribute to the regulation of proinflammatory mediators. In particular, MMP-3 (stromelysin-1) is able to cleave and activate other MMPs such as collagenases [31] and the cytokines TNF*α* and IL-1*β*. In addition, MMPs such as MMP-3 can induce macrophage secretion of TNF*α* leading to cyclooxygenase-2 induction and PGE_2_ production which in turn increases MMP expression. This nexus between MMPs and prostanoids may be relevant in the pathogenesis of chronic inflammatory diseases and cancer [32].

There are a number of ways in which MMP-3 participates in tissue destruction. Studies in MMP-3 knockout mice have shown the key role of this MMP in cartilage lesion and the contribution of synovial macrophages to MMP-mediated damage [33]. We have previously shown that HO-1 overexpression downregulates MMP-3 and other degradative enzymes in articular cells such as synoviocytes [5]. In the current study, we have demonstrated that deficiency in myeloid HO-1 enhances the induction of MMP-3 by zymosan *in vivo*, which may contribute to the inflammatory amplification process involving cytokines, eicosanoids, and proteases which results in tissue damage.

Interestingly, HO-1^M-KO^ mice showed high levels of serum MMP-3 even in the absence of zymosan treatment, which indicates the presence of a systemic inflammatory response in these animals. In fact, serum MMP-3 levels are related to inflammation and tissue damage in conditions such as rheumatoid arthritis, where synovial macrophages are crucial in early MMP activity and serum MMP-3 may be a biomarker of disease activity and predictor of joint destruction [34, 35]. Therefore, our data suggest that myeloid HO-1 contributes to regulatory mechanisms in systemic inflammation.

In conclusion, this study has revealed that myeloid HO-1 mediates protection against the inflammatory response to zymosan *in vivo*. These data point to new directions in therapeutic interventions that may potentially limit the deleterious effects of excessive inflammation.
